# Distinguishing persistence from deviation in composite policy indices: A diagnostic framework for AI readiness trajectory screening and stratification

**DOI:** 10.1371/journal.pone.0355152

**Published:** 2026-11-04

**Authors:** Abedallah Zaid Abualkishik, Adel Khelifi, Ibtesam Mohammed Alawadhi, Ali A. Alwan, Mohamed Elhoseny

**Affiliations:** 1 American University in the Emirates, Dubai, United Arab Emirates; 2 Abu Dhabi University, Abu Dhabi, United Arab Emirates; 3 Dubai Police Academy, Dubai, United Arab Emirates; 4 School of Theoretical and Applied Science, Ramapo College of New Jersey, Mahwah, New Jersey, United States of America; 5 College of Computing and Informatics, University of Sharjah, Sharjah, United Arab Emirates; IULM: Libera Universita di Lingue e Comunicazione, ITALY

## Abstract

Composite indices of national capability reduce dynamic trajectories to static rankings, offering no mechanism to distinguish expected persistence from departures that warrant diagnostic attention. This study asks when a year-to-year change in a national AI-readiness score warrants additional analytical attention. We develop a five-step diagnostic framework (Expect, Detect, Explain, Contextualise, Interpret & Investigate) and apply it to the Oxford Government AI Readiness Index (118 countries, 2021–2024) across two transition windows. An autoregressive baseline explains 97.9% of the variation in next-year scores (W1 in-sample R² = 0.9790, MAE = 1.6506), establishing persistence as the dominant structure and residuals as the primary analytical unit; under a common held-out protocol no flexible specification improves on that baseline, so pillar disaggregation is used for attribution rather than prediction. Government is the pillar most frequently selected by the attribution rule among flagged deviations, with wide uncertainty and a threshold-sensitive ranking (50.0% in W1, 45.8% in W2 after normalisation; Wilson 95% CI 31–69% in W1). Regime analysis defines three Level-dominated readiness strata (k-means, k = 3; W1 trajectory stability ratio, TSR = 0.9407); the W1 directional differentiation in ΔLevel (p = 0.0020) is consistent with mean reversion under the below-unity AR(1) slope (0.954), with the regime term non-significant once continuous prior score is controlled (ANCOVA p = 0.389), and the W2 non-recurrence (p = 0.3643) coincides with a slope near unity (1.007). The regime typology therefore serves as an interpretive stratification device rather than an independent source of trajectory differentiation. Zero skip-a-level movements occur in both windows, consistent with strong persistence rather than independent evidence of ordered mobility. By screening departures from expected persistence using a signed-decile rule with window-specific cut-points (24 of 118 observations flagged per window by construction), the framework reconceptualises composite indices as state-dependent diagnostic systems and supports targeted follow-up monitoring.

## 1. Introduction

National AI readiness indices have become central instruments of government strategy, international benchmarking, and development policy. The Oxford Government AI Readiness Index (GARI) [1–4] aggregates government, technology-sector, and data and infrastructure pillars into annual country scores, while the Stanford AI Index [5] tracks AI development more broadly. Governments increasingly formulate national AI strategies [6,7], and related research links AI capability and adoption to public governance and policy [8,9], public-sector performance and digital service capacity [10,11], and competition policy [12]. In this study readiness indices are treated as diagnostic instruments for researchers and organisations using the index as a readiness proxy, rather than as direct triggers for government decisions.

Their influence rests on an interpretive practice the measurement literature has not validated: reading year-on-year composite score changes as direct evidence of capability progress or decline. Composite indices measuring slowly evolving institutional capacities exhibit strong autoregressive persistence, so most variation in next-period published scores is strongly conditioned by the prior score; without a model of expected trajectory, analysts cannot distinguish routine fluctuation from departures that warrant attention. Composite indices are also sensitive to modelling assumptions and often mask structural heterogeneity across countries [13–17]. Rankings that are fragile by construction and blind to dynamics offer a weak basis for the strategic decisions they are routinely asked to support.

Existing deviation-based work is either cross-sectional or typological: Mandon [17] identifies overperformers cross-sectionally against an economic-complexity baseline (IMF AIPI), and Abualkishik et al. [18] established a trajectory typology. The complementary problem, separating single-period departures that warrant diagnostic attention from persistence-driven noise within a temporal baseline, remains unaddressed and is the focus here.

This paper addresses that gap with a short-horizon diagnostic framework that reframes AI readiness measurement from a static ranking exercise into a structured screening problem: the residual, not the score, is the diagnostic unit, a principle that may apply to other composite measurement systems with strong autoregressive structure. Applied to the Oxford GARI for 2021–2024 across 118 countries, the framework proceeds through five steps: modelling expected persistence, detecting deviations, attributing them to the leading normalised pillar change, contextualising them within readiness regimes, and generating regime-conditioned follow-up priorities, using temporally ordered modelling designed for short panels where long-run inference is infeasible. The panel provides two consecutive transition windows (2022 → 2023 and 2023 → 2024), enabling a temporal consistency check that a single window cannot support. The framework is diagnostic and non-causal by design: its purpose is screening departures from expected persistence, not mechanism estimation.

The study asks when a year-to-year change in a national AI-readiness score warrants additional analytical attention. The analysis is guided by three research questions:

RQ1: How strongly do published AI-readiness scores persist year to year, and which country-year observations fall outside the signed-decile screening rule?

RQ2: Among flagged observations, which normalised pillar change is largest, and how sensitive are attribution patterns to the screening rule?

RQ3: How are flagged observations and short-horizon transitions distributed across readiness strata, and which patterns recur across consecutive transition windows?

The study makes four contributions. First, it redefines composite indices as short-horizon persistence systems and centres interpretation on departures from expected persistence rather than score levels alone. Second, it applies trajectory-deviation logic from the latent change score and panel-data literature to composite AI readiness indices, adapting standard methods to short composite panels where collinearity and sample depth preclude direct SEM application. Third, it delivers an integrated diagnostic framework combining persistence modelling, normalised pillar attribution, and regime-conditioned contextualisation in a replicable procedure: the integration and the follow-up mapping layer constitute the methodological contribution, not the individual components. Fourth, it illustrates regime-conditioned follow-up insights across two consecutive transition windows, providing a two-window consistency check of stratum mobility for the Oxford GARI and extending the practical utility of composite indices in data-limited environments.

The practical consequence is direct. The autoregressive baseline explains 97.9% of W1 score variation, and the signed-decile rule leaves 79.7% of observations unflagged by construction. The framework therefore treats annual score movements first as a screening problem: identify observations outside the chosen band, determine which normalised pillar change is largest, and interpret the case within its readiness stratum. The rest of the paper is organised as follows: Section 2 presents the conceptual framework; Section 3 reviews the literature and identifies four gaps; Section 4 describes the methodology; Section 5 reports results; Section 6 discusses implications; Section 7 concludes.

## 2. Conceptual framework

This study is organised around a single diagnostic question: when a country’s AI readiness score changes year-to-year, does it reflect expected persistence or a notable departure requiring diagnostic attention? If the latter, which pillar changed most, and how should it be interpreted within its readiness stratum? Fig 1 presents the framework as a five-step model; each step is defined below and operationalised in Section 4.

**Fig 1:**
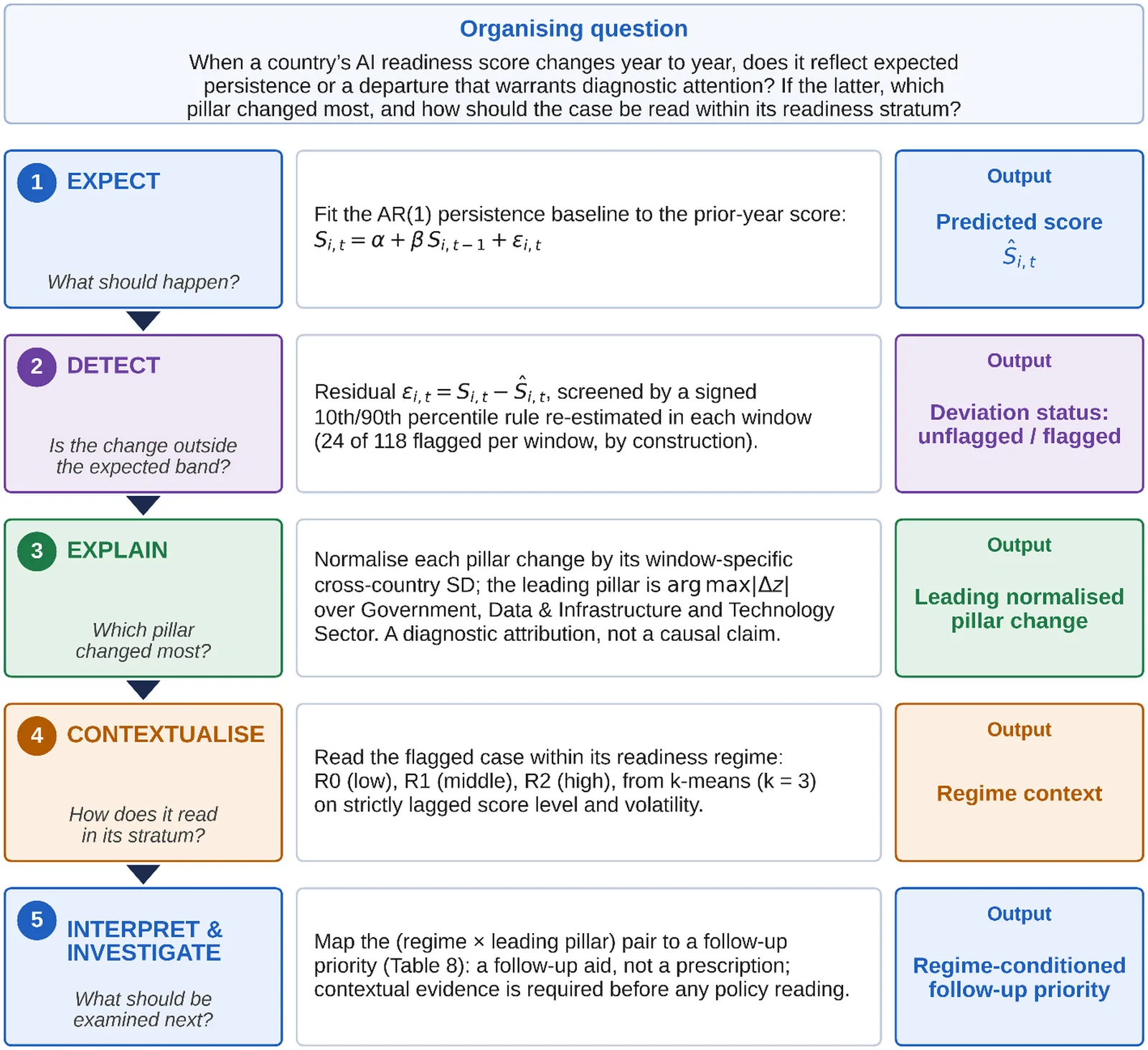
Five-step diagnostic framework for AI readiness trajectory analysis.

### 2.1. Step 1: Expect: Establishing the persistence baseline

A trajectory diagnosis starts from a model of the score expected under the fitted short-horizon persistence pattern. AI readiness scores exhibit strong short-horizon persistence, a plausible pattern for composite indices measuring slowly changing institutional and infrastructural capacity [19–21]. Step 1 represents this as a persistence baseline in which the predicted score for period t is a function of the prior-period score (model form in Section 4.4), yielding the predicted score ${\hat{S}}_{t}$: the fitted persistence expectation.

### 2.2. Step 2: Detect: Measuring the deviation

The residual $\varepsilon_{t}=S_{t}-{\hat{S}}_{t}$ is the framework’s diagnostic unit. A small residual, within the expected range of variation, indicates the score changed as persistence anticipated; a large residual indicates a departure the prior-period score did not predict and warrants additional analytical attention [22]. The output is a deviation status: unflagged, or flagged for attribution.

### 2.3. Step 3: Explain: Identifying the leading normalised pillar change

When an observation is flagged, Step 3 asks which pillar changed most. The GARI composite is built from Government, Technology Sector, and Data & Infrastructure pillar scores [1–3], so a deviation from composite persistence can be examined through the pillar changes behind it. Each pillar’s year-on-year change is normalised by the window-specific cross-country standard deviation of that pillar’s annual change, and the pillar with the largest normalised absolute change is designated the leading normalised pillar change: a diagnostic attribution, not a causal claim [15,23].

### 2.4. Step 4: Contextualise: Interpreting deviation within regime

The same deviation does not carry the same meaning for all countries: constraints, bottlenecks, and interpretive implications differ across the readiness distribution. Step 4 interprets the deviation within one of three readiness strata, R0 (low), R1 (middle) and R2 (high readiness), defined by clustering countries on their prior score level and score volatility at the baseline period (strictly lagged features; the contemporaneous momentum term is excluded to avoid outcome-period contamination) [21,24]. Whether the regimes exhibit distinct short-horizon patterns is examined empirically in Section 5, with two consecutive transition windows enabling a temporal consistency check.

### 2.5. Step 5: Interpret & Investigate: Generating regime-conditioned follow-up priorities

Step 5 translates the diagnosis into follow-up priorities conditioned jointly on the leading pillar change (Step 3) and the readiness stratum (Step 4) [17]: an R0 flagged case with Government as the leading normalised pillar change sits differently from an R2 case with Technology as the leading change, even at identical residual magnitudes. The (pillar, stratum) pair maps to a follow-up priority (what to examine next, not an intervention to adopt), with the underpinning evidence developed in Section 5 and operational rules in Section 6 (Algorithm 1).

### 2.6. The three analytical foundations

The five-step framework rests on three analytical foundations, introduced here and operationalised in Section 4.

**Persistence baseline.** Path-dependence theory, in which prior institutional configurations constrain subsequent trajectories through increasing returns and capability lock-in [19,21], motivates the expectation of strong prior-state dependence in AI readiness scores and the prior-period score as the primary predictor (validated in Section 5.1).

**Normalised pillar attribution.** The three pillars’ annual changes differ markedly in variance, so each pillar’s annual change is normalised by the window-specific cross-country standard deviation of that pillar’s annual change before comparison, placing the pillars on a common unit (parameters in Section 4.4).

**Regime stratification.** Countries at different readiness levels face structurally different constraints and bottlenecks [24]; a uniform diagnostic would confound them. Grouping countries on lagged score level and volatility enable regime-conditioned interpretation of deviations (clustering design in Section 4.3).

## 3. Literature review

### 3.1. Composite benchmarking: Scope and validity

AI readiness measurement is motivated by research linking AI adoption to government performance [10,11], public governance [8,9], and competition policy [12]. The Oxford GARI [1–4] and the Stanford AI Index [5] have become prominent reference points in national AI policy debate [6,7], an influence that exceeds their analytical foundations. Methodologically, composite rankings are sensitive to index design. Miškufová et al. [14] find statistically significant differences in country rankings across four digital-competitiveness indices built on different methodological frameworks, although the rankings remain strongly concordant overall; Nasution et al. [15] re-assess the Oxford GARI ranking using fuzzy LMAW weights and non-linear aggregation; and Hötte et al. [25] show that four patent-based definitions of AI agree on only 1.37% of patents, so measurement choices shape what is counted as AI. What composites aggregate, they also obscure.

Normatively, Nzobonimpa and Savard [16] find that high GARI scores often coexist with weak responsible AI governance. Mandon [17] shows that within score bands countries vary substantially relative to structural expectations; this static analysis does not examine whether heterogeneity evolves over time, a gap addressed here. Silva et al. [13] show for the Network Readiness Index that indicators differ markedly in their influence on the NRI’s own economic and social impact pillars, underlining the value of examining index structure rather than headline rankings alone. Taken together, these findings show that composite rankings are methodologically sensitive, normatively incomplete, and temporally blind: the three limitations motivating this study’s diagnostic reframing.

### 3.2. AI readiness as a path-dependent process

Composite scores exhibit autoregressive continuity, consistent with accumulated institutional and infrastructural capacity. Pierson [19] provides the theoretical foundation through state dependence, where prior institutional configurations constrain trajectories via increasing returns and lock-in; Brekke et al. [20] confirm this empirically for digital transformation; Lundvall [21] identifies the self-reinforcing complementarities across R&D, education, and industry. This motivates the persistence baseline in Step 1: most variation is expected, and deviations are the quantity screened. These works do not, however, predict ordered, no-leapfrogging transitions; that adjacency is a descriptive property of the measurement space (Section 5.4). The Level–Momentum–Stability triad used here was formalised in our prior work [18]; regime construction here uses its strictly lagged {Level, Volatility} subset; signed momentum enters only the contemporaneous comparison variant and the descriptive regime profiles, not the score-prediction models (M1–M3).

At the governance level, Horwitch [6] and Mah [7] discuss how national AI strategies are implemented, and van Noordt and Tangi [26] find that public-sector AI capability rests on tangible, human and intangible resources, with the capability to implement lagging the capability to develop. This literature provides framing only; path dependence is not tested causally here.

### 3.3. Structural heterogeneity and multi-dimensional readiness

AI readiness is inherently multi-dimensional: different constraints bind at different levels. Tangi et al. [27] document that public-sector AI uptake depends on organisational capacity, expertise, collaboration and data governance, and Jöhnk et al. [28] identify multiple technical and organisational categories of AI readiness. These findings motivate pillar-level decomposition in Step 3: a composite cannot reveal which dimension constrains a trajectory. At the organisational level, Jöhnk et al. [28], Borges et al. [29], and Dwivedi et al. [9] show that AI adoption and readiness depend on multiple organisational and strategic conditions; related context dependence is documented in digital-transformation maturity research [30]. Composite scores aggregate what they cannot disentangle. At the cross-national level, Cirera et al. [24] show that the constraints on technology adoption are context-dependent rather than uniform across countries. This heterogeneity motivates regime stratification in Step 4: identical deviations carry different meaning across readiness levels, and a uniform diagnostic could misread them.

### 3.4. Machine learning as a diagnostic instrument

Machine learning is used here as a predictive comparator only in the held-out evaluation (Section 5.1.1), where it does not outperform AR(1), and otherwise as a descriptive complement: deviations are detected from AR(1) residuals and attributed by a normalised pillar-change rule, while Random Forest importance summarises pillar structure descriptively. Its use is motivated by two limitations of regression in composite settings: OLS models including a composite and its pillars as regressors face near-perfect structural collinearity, rendering coefficients uninformative, and linear models cannot capture heterogeneous relationships across development levels.

Pramanik et al. [23] demonstrate the feasibility of using XGBoost and explainable-AI methods to study cross-national AI-readiness enablers. Residual analysis provides a standard means of examining departures from fitted expectations [22]. Here that logic is adapted operationally: the AR(1) residual is the quantity screened for departures from short-horizon persistence, not evidence of structural change in itself.

The logic of detecting deviations from expected trajectories given prior state is well established: latent change score models [31] and random-intercept cross-lagged panel models [32] model expected change from prior state and treat residuals as departures from fitted expectations. This setting precludes direct SEM application: collinearity makes the coefficients degenerate, short panels (3–4 years) prevent full latent modelling, and cross-country heterogeneity requires stratification rather than pooled estimation. The contribution here is therefore methodological translation and applied integration, not procedural novelty.

### 3.5. Synthesis: Four analytical gaps

The strands above show that AI readiness measurement is policy-consequential, methodologically fragile, strongly persistent, structurally heterogeneous, and amenable to ML-based diagnostics. Four gaps remain.

**Gap 1:** No residual audit for composite persistence failure. Nasution et al. [15] examine how GARI results change under alternative weights. This study instead asks where the existing composite fails to predict the next period and which normalised pillar change explains that failure, a complementary but structurally distinct question not addressed for the Oxford GARI.

**Gap 2:** No held-out developmental alignment check. Miškufová et al. [14] compare composites with other composite instruments, a within-family approach that cannot detect shared biases. This study tests whether the Oxford composite score aligns with World Bank income classifications under five-fold cross-validation, providing an external reference.

**Gap 3:** No temporal within-stratum variance analysis. Mandon [17] documents within-band heterogeneity at a single point in time. This study tests whether that heterogeneity increases or decreases across periods and whether the direction varies systematically across the readiness distribution.

**Gap 4:** No regime transition description with inferential transparency. To our knowledge, no prior study describes Oxford GARI transition structure using a full count matrix, Wilson confidence intervals, and a marginal-preserving permutation benchmark. Our prior typology [18] found that switching across readiness strata is uncommon; here transition structure is examined window by window rather than typologically.

## 4. Materials and methods

This study employs a temporally ordered, non-causal diagnostic framework. In the primary specification, all predictors are constructed from information available at period t − 1 and all outcomes are evaluated at period t, preventing forward-looking information from entering the analysis; the contemporaneous comparison variant (Section 4.3), used only in analyses identified as such, is the sole exception.

### 4.1. Data sources and panel construction

The primary data source is the Oxford GARI for 2021–2024 [1–4], providing annual composite readiness scores (0–100) and Government, Technology Sector, and Data & Infrastructure pillar scores. World Bank World Development Indicators are used only in the M3 specification. Worldwide Governance Indicators (2021–2023) enter M3 only, as lagged predictors, while World Bank structural indicators cover 2021–2024.

Panel construction applies three rules (removing indicators below 80% availability, excluding country-year observations below 70%, and dropping countries without full four-year coverage), yielding 118 countries, 472 country-year observations, and two transition windows: 2022→2023 (W1) and 2023 → 2024 (W2). GARI is re-methodologised across editions (42 indicators and 160 countries in 2021, 39 and 181 in 2022, 39 and 193 in 2023, 40 and 188 in 2024 [1–4]), so the windows are not measurement-invariant; consistent with this, the share of countries rising differs (W1: 78/118; W2: 98/118), and the W2 shift may partly reflect recalibration.

### 4.2. Feature construction

Four features are defined to capture position, direction, and stability in the measurement space; the primary analysis uses two of them, Level and Volatility. *Level* represents the prior-period composite score and serves as the primary predictor, defined as ${\text{Level}}_{i,t}=S_{i,t-\text{1}}$. Two momentum measures are defined; neither enters the score-prediction models (M1–M3). The prior-period momentum, reported descriptively in the regime profiles (Section 5.2.1), is ${\text{Momentum}}_{i,t}^{\text{pred}}=S_{i,t-\text{1}}-S_{i,t-\text{2}}$

The contemporaneous momentum is defined as ${\text{Momentum}}_{i,t}^{\text{cont}}=S_{i,t}-S_{i,t-\text{1}}$, and is used only in the contemporaneous comparison variant of the regime clustering (Section 4.3); it plays no part in the primary lagged regime construction. *Volatility* captures score stability and is defined as the standard deviation of the two most recent prior scores, ${\text{Volatility}}_{i,t}=\sigma \left(S_{i,t-\text{2}},S_{i,t-\text{1}}\right)$. With two scores this equals the absolute prior-period momentum divided by √2, so the primary features carry its magnitude but not its sign.

### 4.3. Regime construction and the trajectory stability ratio

Readiness strata (referred to as regimes throughout for continuity) are defined by k-means clustering on strictly lagged features {Level, Volatility} (primary specification; the contemporaneous {Level, Momentum, Volatility} variant is retained only for comparison) at a baseline year. Centroids are estimated on the baseline cross-section and applied without re-estimation to subsequent periods, preserving prospective assignment.

Three regimes are retained for interpretability (low/middle/high strata). The Trajectory Stability Ratio is defined as ${\text{TSR}}_{w}=\frac{\text{1}}{N}\sum_{i=\text{1}}^{N}1\left[R_{i,t}=R_{i,t-\text{1}}\right]$, the share of countries whose stratum assignment is unchanged across window w; per-stratum retention is the same quantity restricted to countries with $R_{i,t-\text{1}}=r$, and the two are reported separately throughout. TSR is reported descriptively, not as a selection criterion, and k = 3 is an interpretability choice rather than a geometric optimum (comparison-variant k-sweep and internal validity indices in Section 5.5; no sweep is reported for the primary specification because its partition is equivalent to banding on Level, so TSR would rise mechanically as k falls). Clustering uses k-means (n_init = 20, random_state = 42) on unstandardised features, so Level dominates the metric by design.

Because contemporaneous momentum may introduce circularity, we adopt a strictly lagged specification as primary; agreement between assignments is quantified using the adjusted Rand index, reported in Section 5.5 for the lagged-versus-contemporaneous comparison and for two sensitivity comparisons (Level-only clustering, and clustering on standardised features). Full empirical results are reported in Section 5.

### 4.4. Predictive model specifications

Four specifications, plus a Level-only Random Forest control, are estimated on each transition window (lagged features; next-period outcomes), with W1 (2022 → 2023) as the primary evaluation window and W2 (2023 → 2024) providing a temporal consistency check:


$$\begin{array}{c} {M1(AR(1)baseline):\text{\textasciitilde{}\textasciitilde{}}S}_{i,t}=\alpha +\beta S_{i,t-\text{1}}+\varepsilon_{i,t} \end{array}$$

$$\begin{array}{c} M2(InternalRF):{\text{\textasciitilde{}\textasciitilde{}}S}_{i,t}=f(S_{i,t-\text{1}},Gov_{i,t-\text{1}},Tech_{i,t-\text{1}},Data_{i,t-\text{1}}) \end{array}$$

$$\begin{array}{c} M2b(Pillars-onlyRF):{\text{\textasciitilde{}}S}_{i,t}=f(Gov_{i,t-\text{1}},Tech_{i,t-\text{1}},Data_{i,t-\text{1}}) \end{array}$$

$$\begin{array}{c} M3(FullRF):{\text{\textasciitilde{}}S}_{i,t}=f(M\text{2}\text{ features},WB_{i,t-\text{1}}) \end{array}$$

M1 defines the persistence baseline. M2 adds lagged pillars to Level (Random Forest, since composite–pillar collinearity makes OLS unsuitable); M2b uses the pillars without Level; M3 adds 15 external predictors measured at t − 1: ten World Development Indicators (secure internet servers per million people, internet penetration (%), mobile and fixed-broadband subscriptions per 100 people, GDP per capita, GDP growth (%), industry value added (%), high-technology exports (%), inflation (%) and female labour-force participation (%)) and five Worldwide Governance Indicators (rule of law, government effectiveness, regulatory quality, control of corruption and political stability), with exact definitions in the reproducibility package. A Level-only Random Forest isolates the effect of model flexibility. Linear Regression, LASSO and Gradient Boosting use the M2 inputs, and a no-change benchmark (${\hat{S}}_{t}=S_{t-\text{1}}$) is also reported.

Held-out MAE is evaluated by 5 × 5 repeated cross-validation with folds shared across specifications, and model differences by a paired country-level bootstrap (B = 2000). AR(1) residuals are flagged using signed 10th/90th percentile thresholds (Section 5.1.2). Residual thresholds and pillar standard deviations are re-estimated in each window, while regime centroids stay fixed at the 2022 baseline (Section 4.3), so W2 is a temporal consistency check rather than a prospective validation. The leading normalised pillar change is ${\text{arg}\;\text{max}}_{p}\left|\Delta P_{p}/\sigma_{p}\right|$, where $\sigma_{p}$ is the window-specific cross-country standard deviation of pillar p’s annual change. Random Forest models use 500 trees, Gradient Boosting 200 estimators and LASSO α = 0.1; all stochastic procedures use random seed 42. Analyses used Python 3.13; exact package versions are listed in the package README.

### 4.5. Developmental alignment check

A multinomial logistic classifier predicts World Bank income group membership from the 2022 composite score (five-fold stratified cross-validation against a 37.3% majority baseline), testing whether composite score levels align with the World Bank income classification. The classification is the one retrieved on 22 September 2026 and fixed in the analysis code, so this is an external consistency check rather than a contemporaneous 2022 validation.

### 4.6. Regime-anchored pattern analysis

Three trajectory dimensions are anchored to each country’s regime at the start of each window (2022 for W1; 2023 for W2): ΔLevel, TSR, and within-regime score variance change. Cross-regime differences in ΔLevel are assessed per window with the Kruskal–Wallis H test and, with continuous prior score controlled, an ANCOVA partial F test for the regime term given Level, together with the reversion-implied regime means from the fitted AR(1); this helps distinguish regime structure from mean-reversion artefacts. Within-regime variance changes are tested using Levene’s and Fligner–Killeen tests; because the same countries are measured before and after, both are reported for continuity only and a country-level paired bootstrap (B = 2000) supplies the interval used for inference.

### 4.7. Transition description

Transition behaviour is characterised per window by a full count matrix with row-normalised probabilities, per-regime Wilson 95% confidence intervals for retention, and a one-tailed permutation test (10,000 iterations) for the probability of zero or fewer skip-a-level transitions under random regime reassignment, with $p=\frac{b+\text{1}}{B+\text{1}}$, where b of the B permutations are at least as extreme, so that no simulated p-value is reported as zero. Reassignment preserves the observed marginal regime distribution (regime sizes fixed; only country-to-regime mappings shuffled), and findings are reported separately per window. Regime transition probability is additionally modelled as:


$$\begin{array}{c} P(R_{i,t}|Level_{i,t},Volatility_{i,t}) \end{array}$$

Using Random Forest. This is fitted on the contemporaneous comparison-variant regimes and serves as a structural consistency check: regime membership is largely determined by Level; its in-sample accuracy is uninformative, because a Random Forest fits its own training data, and is not interpreted as predictive capacity.

### 4.8. Robustness

Robustness is assessed along three dimensions. Model robustness: held-out MAE compared across Linear Regression, LASSO, Random Forest, and Gradient Boosting. Structural robustness: the regime typology under k ∈ {2, 3, 4, 5} (comparison variant). Temporal robustness: the two transition windows provide a temporal consistency check, with regime retention stability (the W1–W2 change in TSR) and predictive performance stability (AR(1) held-out MAE by window, Section 5.1.1) reported separately, the former reflecting regime structure and the latter differences across the two observed transition windows.

### 4.9. Mapping framework steps to research questions

Table 1 maps each framework step to its research question and empirical section. Step 5 (Interpret & Investigate) is not tested in Section 5; it is derived from the results and developed in Section 6, where the diagnostic procedure (Algorithm 1) and the follow-up matrix set out how findings are read.

**Table 1:** Mapping of framework steps to research questions and empirical sections.

Framework Step	Function	Research Question	Empirical Section
Step 1: Expect	Establish persistence baseline	RQ1	Section 5.1.1
Step 2: Detect	Identify flagged deviations	RQ1	Sections 5.1.1–5.1.2
Step 3: Explain	Attribute deviation to leading pillar	RQ2	Section 5.1.3
Step 4: Contextualise	Interpret within regime context	RQ3	Sections 5.2–5.4
Step 5: Interpret & Investigate	Generate follow-up priorities	(No RQ; derived from results)	Section 6 (Discussion)

## 5. Empirical results

Results are organised by research question and map to the framework steps in Table 1. RQ1 (Sections 5.1.1–5.1.2) operationalises Steps 1–2, persistence and screening; RQ2 (Section 5.1.3) operationalises Step 3, attribution; RQ3 (Sections 5.2–5.4) operationalises Step 4, stratum context and temporal consistency. Section 5.5 reports robustness checks. Step 5 (follow-up priorities) is not empirically tested but derived in Section 6.

### 5.1. Persistence, screening and attribution

#### 5.1.1. Persistence baseline and model performance.

Composite scores are highly persistent: the prior-period score explains nearly all next-year variation (R² = 0.9790 in W1 and 0.9662 in W2), with AR(1) slopes of 0.954 and 1.007. The AR(1) model therefore defines the diagnostic floor for residual-based analysis, and held-out comparisons show no improvement over it (Table 2; Fig 2).

**Table 2:** Model performance across specifications (W1 and W2).

Model	Features	In-sample MAE	Held-out CV MAE (SD)	Interpretation
W1 (2022 → 2023)				
M1	Level only (AR(1))	1.6506	1.6830 (0.0208)	Baseline; barely overfits (1.6506 → 1.6830)
M2	Level + Pillars (RF)	0.7478	2.0992 (0.0688)	In-sample gain does not survive validation
M2b	Pillars only, no Level (RF)	0.8368	2.2493 (0.0458)	In-sample gain does not survive validation
M3	M2 + World Bank indicators	0.7639	2.1507 (0.0531)	No gain over M2; inferior to the baseline out of sample
RF control	Level only (RF)	0.8221	2.1393 (0.1025)	Shows a similarly large in-sample gain without pillar information
Linear Regression	Level + Pillars (linear)	1.5919	1.6858 (0.0258)	Tracks the baseline
LASSO	Level + Pillars (linear, α = 0.1)	1.6127	1.6855 (0.0342)	Tracks the baseline
Gradient Boosting	Level + Pillars	0.1853	2.3622 (0.1004)	Largest in-sample gain; weakest held-out
No-change	Prior score carried forward	—	1.8009 (no fitting)	No fitting; ignores the average upward drift (+0.887)
W2 (2023 → 2024)				
M1	Level only (AR(1))	2.1956	2.2402 (0.0291)	Baseline; barely overfits (2.1956 → 2.2402)
M2	Level + Pillars (RF)	0.9776	2.6607 (0.1022)	In-sample gain does not survive validation
M2b	Pillars only, no Level (RF)	1.0159	2.7664 (0.1405)	In-sample gain does not survive validation
M3	M2 + World Bank indicators	1.0079	2.7847 (0.1050)	No gain over M2; inferior to the baseline out of sample
RF control	Level only (RF)	1.1204	2.9459 (0.0320)	Shows a similarly large in-sample gain without pillar information
Linear Regression	Level + Pillars (linear)	2.1809	2.2922 (0.0321)	Tracks the baseline
LASSO	Level + Pillars (linear, α = 0.1)	2.1775	2.2594 (0.0278)	Tracks the baseline
Gradient Boosting	Level + Pillars	0.2075	2.8596 (0.2176)	In-sample gain does not survive validation
No-change	Prior score carried forward	—	2.8504 (no fitting)	No fitting; ignores the average upward drift (+2.247)

Both columns use the same 118-country sample; held-out values come from 5 × 5 repeated cross-validation with folds identical across specifications (RF 500 trees, seed 42), with repeat-level SDs in parentheses. The no-change benchmark involves no fitting, so only its out-of-sample MAE is shown. Paired country bootstrap of out-of-fold MAE differences (B = 2000; 95% CI): M2 − M1 + 0.4161 [+0.240, + 0.621] in W1 and +0.4205 [+0.167, + 0.669] in W2; M2 − RF control −0.0401 [−0.215, + 0.131] and −0.2851 [−0.540, −0.038]; M2b − RF control +0.110 [−0.150, + 0.385] and −0.179 [−0.535, + 0.181].

**Fig 2:**
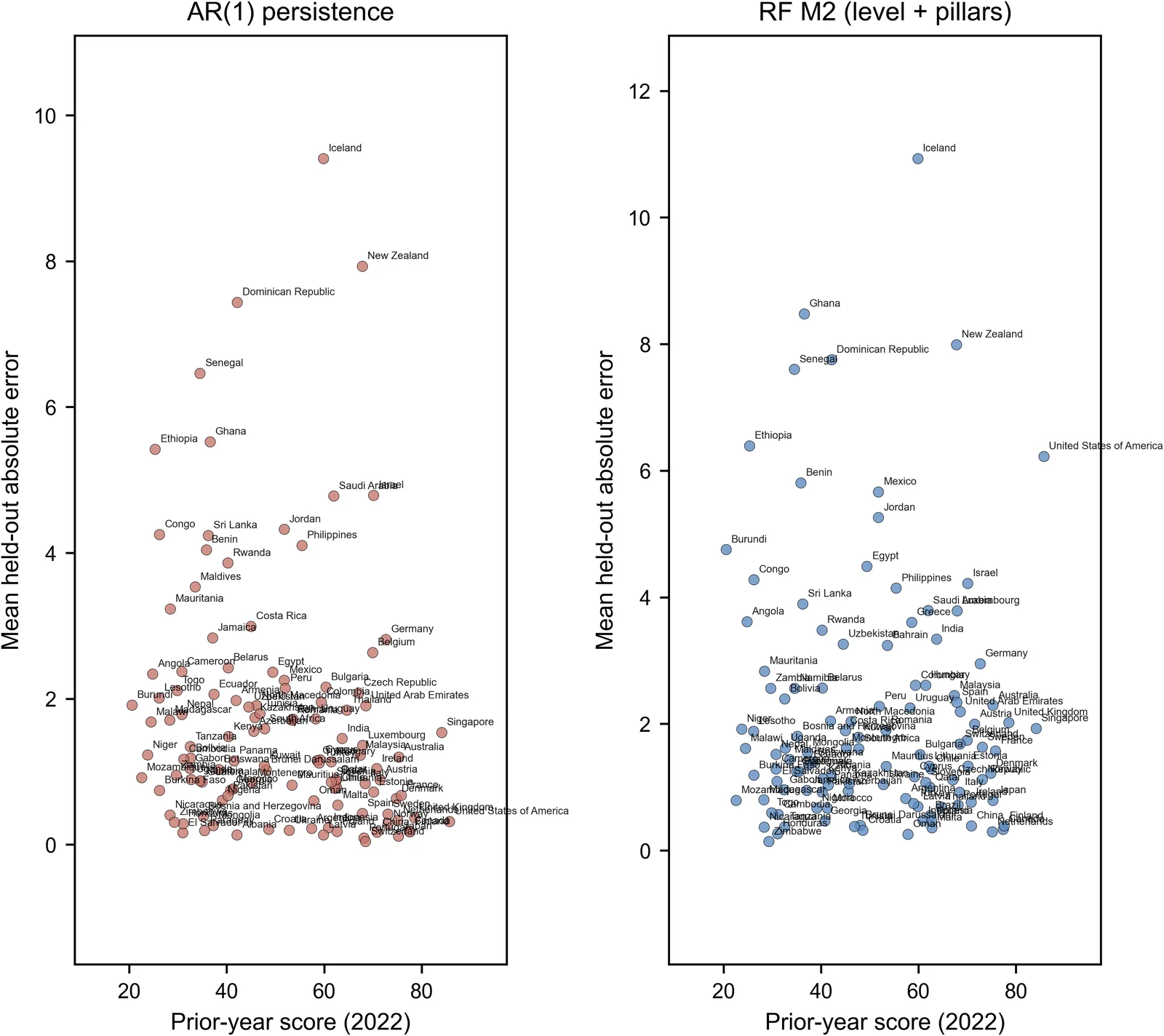
Country-level held-out mean absolute error: AR(1) and RF M2 in W1 under 5 × 5 Repeated Cross-Validation.

Under held-out evaluation the in-sample ordering reverses: no specification improves on the AR(1) baseline in either window, and the baseline barely overfits (Table 2). The paired bootstrap places AR(1) ahead of M2 in both windows; lagged pillars improve on a Level-only forest only in W2, a gain that is not independent information because the composite is built from the pillars, and M2b does not differ reliably from that forest (Table 2 note). Pillar disaggregation is therefore used for attribution, not forecasting. The no-change benchmark also trails AR(1), because it ignores the average upward drift.

#### 5.1.2. Residual structure and diagnostic thresholds.

Shapiro–Wilk rejects normality of the AR(1) residuals (W1 p < 0.0001; W2 p = 0.0002), motivating empirical percentile thresholds; Breusch–Pagan (Koenker variant) does not reject homoskedasticity in W1 (p = 0.524) but rejects it in W2 (p = 0.017), noted as a limitation. A flagged deviation is a signed residual beyond the 10th/90th percentile band (W1 thresholds +2.455 and −2.041), yielding 24 cases per window, the set used for pillar attribution. W2 residuals are more dispersed (SD = 3.0493; thresholds +3.485 / −3.368). Signed percentiles capture acceleration and deceleration separately rather than pooling them under a single absolute cut-off, and thresholds are dataset-specific realisations of a general percentile rule: new applications recompute them from their own residual distributions. Two signed diagnostic categories partition the 118 observations: unflagged observations, for which the rule generates no deviation flag, and flagged deviations, which trigger attribution. Thresholds, counts, and the regime breakdown of the 24 primary-threshold cases are reported in Table 3.

**Table 3:** Diagnostic threshold categories and country distribution (W1).

Diagnostic Category	n	R0	R1	R2
Unflagged (within signed-decile band) −2.041 ≤ residual ≤ +2.455	94 (79.7%)	27	24	43
Flagged deviation residual > +2.455 or <−2.041 (signed 10th/90th percentile; primary threshold)	24 (20.3%)	10	7	7

Each window has 24 primary-threshold observations: W1 12 accelerations, 12 decelerations; W2 12 accelerations, 12 decelerations.

By construction, the signed-decile rule flags 24 of 118 observations per window (Table 3). R0 accounts for the largest share of W1 primary-threshold cases, although baseline score volatility is highest in R1; in W2 the flags concentrate in R1 (Table 4). W2 residuals are heteroskedastic, with lower dispersion at high readiness (Table 4 note), so a single global band flags fewer high-readiness cases; the stratum distribution of flags is not variance-adjusted and should not be read as differential instability. The residual rule shares 19 of 24 flags with the same rule applied to raw score changes in W1 and all 24 in W2, as expected with a W2 slope close to unity. M2 importance assigns over 90% to Level in both windows, reflecting composite–pillar collinearity; M2b importance, reported descriptively, is Government-led in W1 (Fig 3) and more balanced in W2 (Government 0.399, Data & Infrastructure 0.304, Technology 0.298). Case-level attribution uses the normalised pillar-change rule (Section 5.1.3).

**Table 4:** Normalised leading pillar attribution by regime and threshold rule (W1 and W2).

Regime / rule	n	Government	Data & Infra.	Tech. Sector	Total
W1 (2022 → 2023), n = 24					
R0	10	6 (60.0%)	2 (20.0%)	2 (20.0%)	10
R1	7	3 (42.9%)	4 (57.1%)	0 (0.0%)	7
R2	7	3 (42.9%)	2 (28.6%)	2 (28.6%)	7
Total	24	12 (50.0%; 31–69%)	8 (33.3%; 18–53%)	4 (16.7%; 7–36%)	24
W2 (2023 → 2024), n = 24					
R0	9	4 (44.4%)	1 (11.1%)	4 (44.4%)	9
R1	13	5 (38.5%)	7 (53.8%)	1 (7.7%)	13
R2	2	2 (100.0%)	0 (0.0%)	0 (0.0%)	2
Total	24	11 (45.8%; 28–65%)	8 (33.3%; 18–53%)	5 (20.8%; 9–40%)	24
W1 (2022 → 2023) — threshold rule					
±2 SD	7	6 (85.7%; 49–97%)	0 (0.0%)	1 (14.3%)	7
Signed deciles (used)	24	12 (50.0%; 31–69%)	8 (33.3%)	4 (16.7%)	24
20th/80th percentile	48	14 (29.2%; 18–43%)	18 (37.5%)	16 (33.3%)	48
W2 (2023 → 2024) — threshold rule					
±2 SD	6	5 (83.3%; 44–97%)	0 (0.0%)	1 (16.7%)	6
Signed deciles (used)	24	11 (45.8%; 28–65%)	8 (33.3%)	5 (20.8%)	24
20th/80th percentile	48	13 (27.1%; 17–41%)	22 (45.8%)	13 (27.1%)	48

Wilson 95% CIs follow each share. The leading pillar is the largest σ-normalised change (Section 4.4), using σGov = 5.496, σTech = 1.742 and σData = 4.211 in W1 and 7.579, 2.850 and 3.882 in W2. W2 counts by stratum are not variance-adjusted (W2 residual SD 3.851, 3.561 and 1.963 in R0–R2; Section 5.1.2), and the R1 and R2 breakdowns rest on small counts and are provisional pending replication.

**Fig 3:**
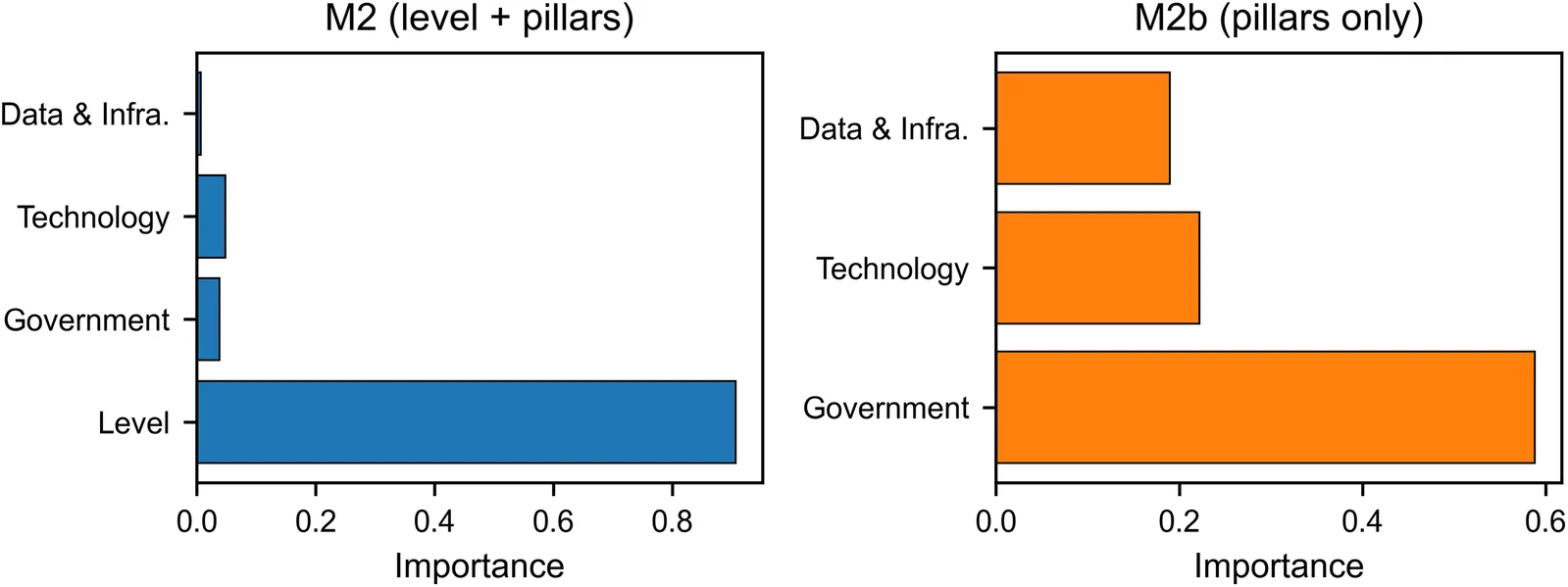
Feature Importance comparison: M2 (Level-Dominated) vs. M2b (Pillars Only).

#### 5.1.3. Attribution of flagged deviations.

Step 3 attributes each of the 24 primary-threshold deviations per window (W1 thresholds in Fig 4) to its leading normalised pillar change (W1 in Fig 5). Two attribution logics are distinguished: M2b RF importance is a descriptive, model-specific summary across all 118 observations, while Step 3 normalised change identifies which pillar changed most in each flagged case; the former is global, the latter case-specific. Table 4 reports the normalised leading pillar by regime.

**Fig 4:**
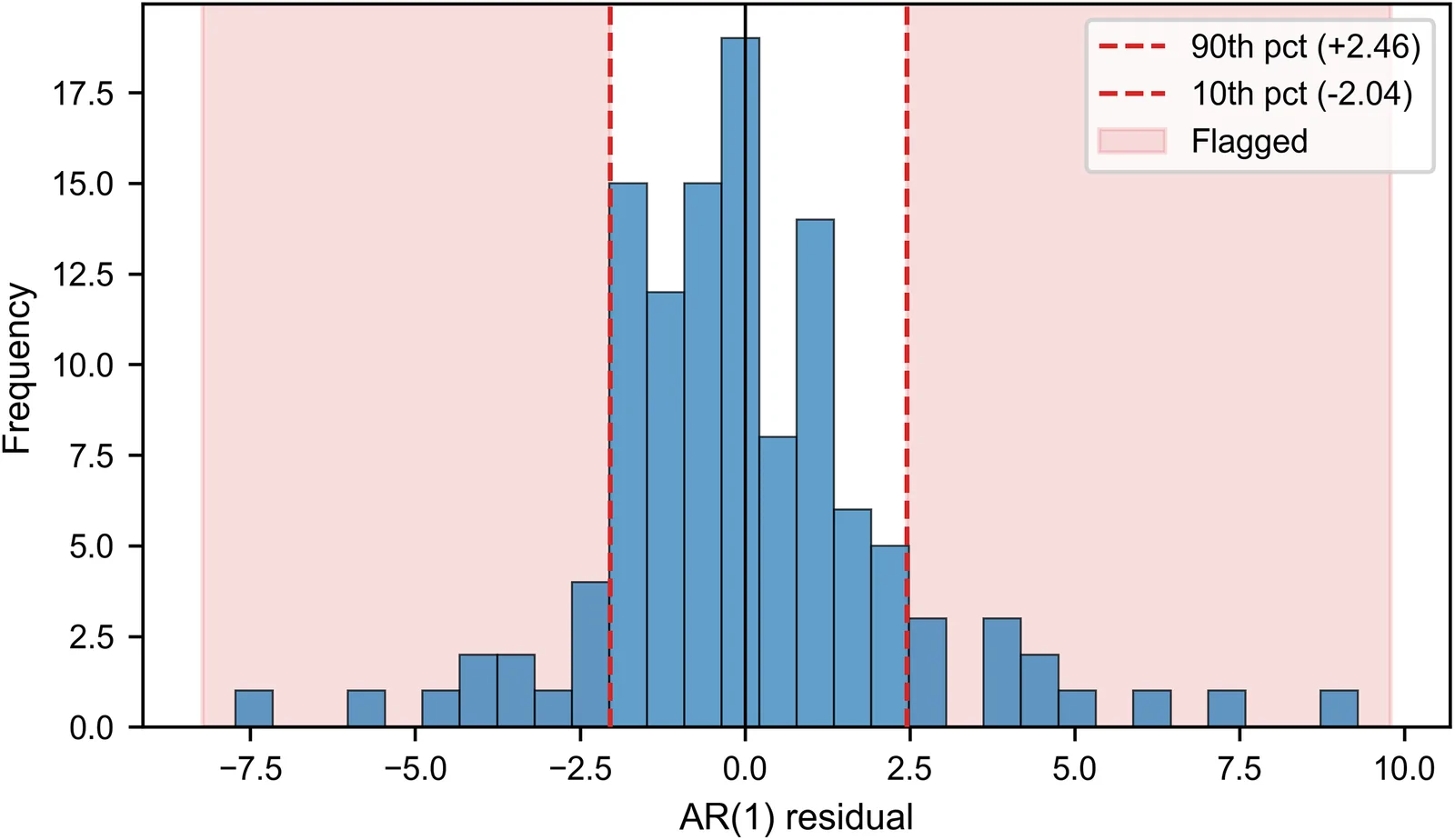
AR(1) Residual distribution with diagnostic thresholds (W1).

**Fig 5:**
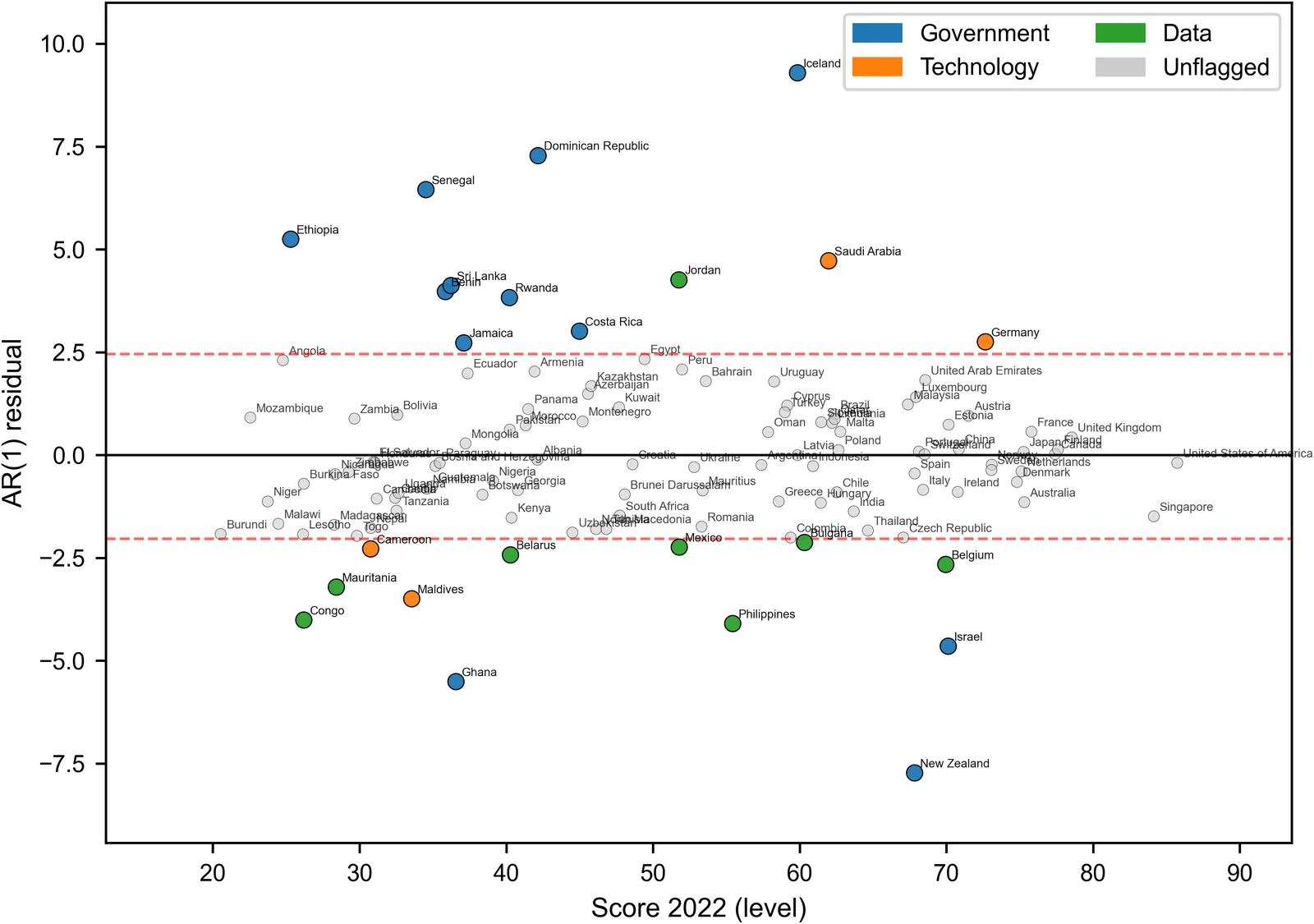
AR(1) Residuals coloured by normalised leading pillar source (W1).

Under the primary rule, Government is the pillar most frequently selected by the attribution rule in both windows, followed by Data & Infrastructure and Technology (Table 4). Signed deciles are a transparent screening rule that does not assume residual normality, but the shares are threshold-sensitive: Government leads more strongly under ±2 SD, whereas Data & Infrastructure leads under a 20th/80th rule (Table 4), so pillar shares are interpreted descriptively rather than as population parameters. σ-normalisation matters: because σGov far exceeds σTech, it reduces Government’s apparent dominance. Technology’s W2 share rises, concentrated in R0 (Table 4) and alongside a wider W2 Technology distribution (Table 4 note); whether this is structural or period-specific requires further windows.

Illustrative W1 cases (Fig 5) include governance-led accelerations in Iceland (R2) and Senegal (R0), with New Zealand (R2) the largest deceleration; in W2 the extremes are Mauritania (R0) and Azerbaijan (R1). The full case list per window is produced by the analysis code deposited in the OSF repository (Data Availability).

### 5.2. Regime structure and developmental alignment

RQ3 opens with the first dimension of Step 4: characterising the structural context in which deviations are interpreted. It addresses whether the three regimes represent distinct readiness strata and, as a validity check, whether composite score levels align with independent developmental classifications.

#### 5.2.1. Regime profiles.

Table 5 provides the interpretive framework for the results that follow, separating mechanical consequences of persistence from diagnostic findings. The framework relies on that persistence: its contribution is identifying where observations depart from the fitted persistence expectation and providing the strata within which those departures are interpreted. The apparent cross-regime differentiation in mean ΔLevel is consistent with a reversion artefact once continuous Level is controlled (Section 5.3.1). The regime structure is visualised in Fig 6. The low linear R² of Level for ΔLevel (Table 6 note) reflects within-stratum scatter rather than absence of mean reversion: the regime means are themselves ordered as reversion implies, so regime grouping should be read as interpretive stratification, not structure beyond score sorting.

**Table 5:** Classification of results: Baseline, mechanical, informative, and exploratory.

Result Type	Finding	Why It Occurs	Interpretation
Baseline	AR(1) persistence (R² = 0.9790 W1; 0.9662 W2)	Common in slowly changing composite indices	Expected baseline; sets diagnostic floor
Mechanical	Under held-out validation no specification improves on the AR(1) baseline (Table 2); the in-sample MAE reduction reflects model flexibility	A Level-only Random Forest, with no pillar information, shows a similarly large in-sample gain	No predictive advantage is claimed; pillar disaggregation is used for attribution (Step 3), not prediction
Mechanical	Random Forest feature importance is descriptive only	Importance is model-specific and computed in sample	Step 3 attribution relies on normalised case-level pillar change, not RF importance
Mechanical	χ² transition significance (χ² = 196.05 W1, 168.48 W2; p < 0.001)	Statistically expected under strong persistence	Threshold only; diagnostic value lies in the pattern
Informative	Government is the pillar most frequently selected by the attribution rule: 50.0% W1, 45.8% W2 (normalised); ranking threshold-sensitive (Table 4)	Not mechanically guaranteed; requires normalised pillar-level change analysis	Shows that flagged deviations are not exclusively technology-led.
Mechanical	Zero skip-a-level transitions in both windows (permutation p = 0.0001)	Consistent with strong persistence (R² = 0.979); the permutation null (28.36 W1, 25.60 W2) is not proximity-constrained and overstates expected skips	Consistent with persistence, not independent evidence of ordered mobility; the regularity recurs in both windows
Mechanical	Directional change differs across regimes in W1 (KW p = 0.0020); not significant in W2 (p = 0.3643)	AR(1) slope below unity (0.954) implies mean reversion across Level-defined regimes; W2 slope ≈ 1 (1.007) removes the reversion	Regime term not significant once continuous Level is controlled (ANCOVA p = 0.389); mechanical, consistent with mean reversion
Exploratory pattern (requires replication)	Within-stratum variance changes: W1 uncertain; W2 R0 (+13.186) and R1 (+18.802) increase; R1 robust to fixed 2022 membership	Paired bootstrap respects repeated-country measurement; Levene and Fligner–Killeen retained descriptively	Exploratory: the W2 R0 and R1 intervals exclude zero, but the comparisons were not a prespecified multiplicity family

**Table 6:** Regime profiles at W1 baseline (2022) and retention in both windows.

Regime	n	Mean Level	Prior change (2021 → 2022)	Volatility	W1 retention [Wilson 95% CI]	W2 retention [Wilson 95% CI]
R0	37	31.0	−1.649	1.820	0.811 [0.658, 0.905]	0.867 [0.703, 0.947]
R1	31	46.3	+0.329	2.246	1.000 [0.890, 1.000]	0.816 [0.666, 0.908]
R2	50	67.4	+0.029	1.706	1.000 [0.929, 1.000]	0.980 [0.895, 0.996]

Prior change is the 2021 → 2022 score change (prior-period momentum, Section 4.2), reported descriptively. W2 retention is computed for the strata at the start of W2 (n = 30, 38 and 50). W1: KW H = 12.402, p = 0.0020; Level R² for ΔLevel = 0.0973; the regime term is not significant once continuous Level is controlled (ANCOVA partial F = 0.95, p = 0.389; Kruskal–Wallis on AR(1) residuals p = 0.319). W2: KW H = 2.019, p = 0.3643; ANCOVA regime term p ≈ 1.0.

**Fig 6:**
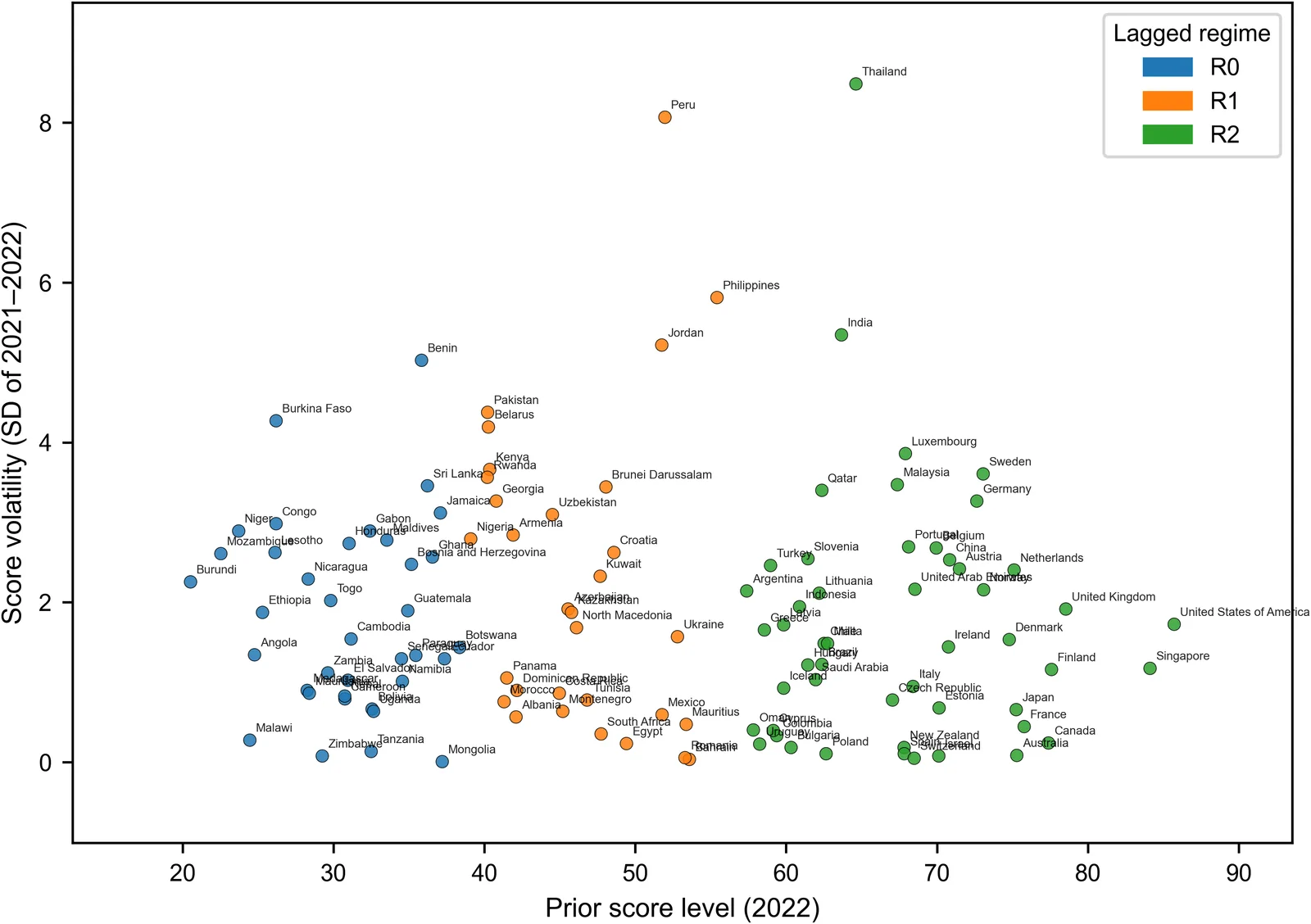
Regime structure in the lagged level–volatility plane.

#### 5.2.2. Developmental alignment.

The multinomial logistic classifier (Section 4.5) achieves cross-validated accuracy of 0.6464 ± 0.1418 against a 37.3% majority baseline. The high fold-level variance (per-fold accuracy 0.500 to 0.913) indicates instability, so results are read as a consistency check rather than a robust alignment claim (Fig 7). Misclassifications are concentrated at adjacent income boundaries, with only two non-adjacent errors in the pooled out-of-fold matrix, indicating the composite tracks developmental structure directionally but imperfectly [14,17].

**Fig 7:**
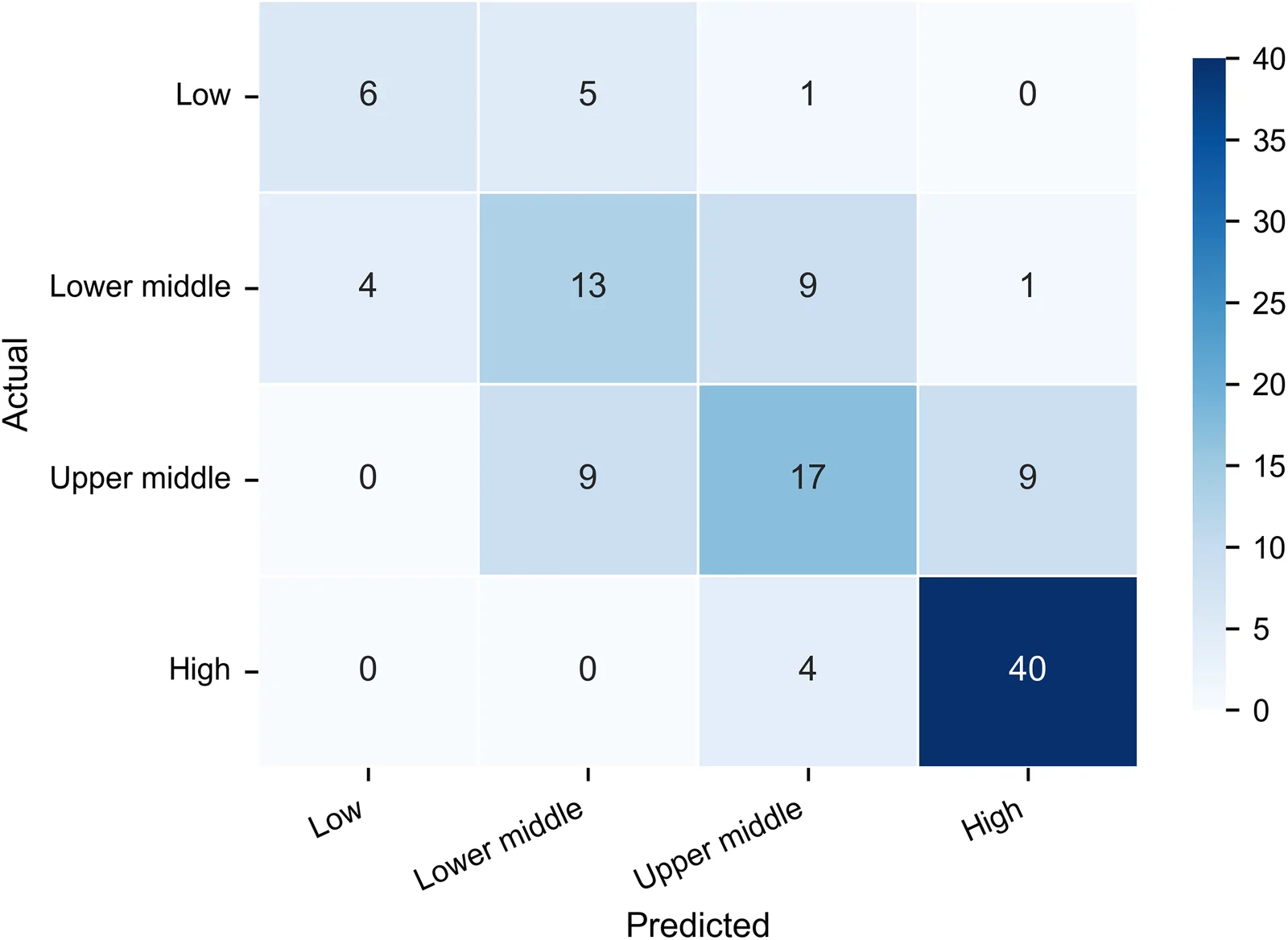
Out-of-fold confusion matrix for developmental alignment with World Bank Income Groups under Five-Fold Stratified Cross-Validation.

### 5.3. Trajectory patterns across regimes

RQ3 continues with the second dimension of Step 4: testing whether trajectory patterns differ systematically across regimes in direction and dispersion. Direction assesses whether regimes exhibit distinct directional change beyond rankings; dispersion assesses whether within-stratum heterogeneity differs between windows.

#### 5.3.1. Directional change.

Directional change differs across regimes in W1 (Kruskal–Wallis p = 0.0020) but not in W2 (p = 0.3643; Table 6 note). The W1 differentiation is consistent with mean reversion: with an AR(1) slope below unity (0.954), reversion toward the fitted fixed point mechanically orders the regime means, the observed means follow the reversion-implied ordering (Table 7 note), and the regime term is not significant once continuous Level is controlled (ANCOVA p = 0.389). The W1 differentiation is therefore classified as mechanical (Table 5). In W2 all regimes improve by similar amounts (Table 7). Given the cross-edition recalibration (Section 4.1) and the near-unity W2 slope (1.007), this is expected: the fitted AR(1) implies almost no separation of regime means, and the observed means match the reversion-implied values (Table 7 note). W1 distributions are shown in Fig 8.

**Table 7:** Regime-level trajectory statistics and within-stratum variance change (W1 and W2).

Regime	n	Observed mean ΔLevel	SD	Variance before → after	Δ variance	Paired bootstrap 95% CI	Levene p	Fligner p
W1 (2022 → 2023)								
R0	37	+1.512	2.456	21.99 → 30.01	+8.020	[−0.040, +15.854]	0.572	0.408
R1	31	+1.446	2.398	24.30 → 23.65	−0.659	[−8.412, +6.980]	0.838	0.940
R2	50	+0.077	2.336	49.80 → 45.38	−4.428	[−10.459, +1.302]	0.789	0.643
W2 (2023 → 2024)								
R0	30	+2.084	3.849	15.98 → 29.17	+13.186	[+2.356, +24.552]	0.171	0.225
R1	38	+2.234	3.567	26.81 → 45.61	+18.802	[+8.093, +29.139]	0.037	0.043
R2	50	+2.355	1.958	45.38 → 46.97	+1.588	[−4.429, +7.934]	0.995	0.979

AR(1)-implied mean ΔLevel for R0, R1 and R2: W1 + 1.78, + 1.08 and +0.11; W2 + 2.09, + 2.21 and +2.37. With membership fixed at 2022, the W2 variance increase is + 9.203 [+0.332, + 18.399] for R0 and +17.864 [+5.742, + 28.984] for R1 (R2 unchanged).

**Fig 8:**
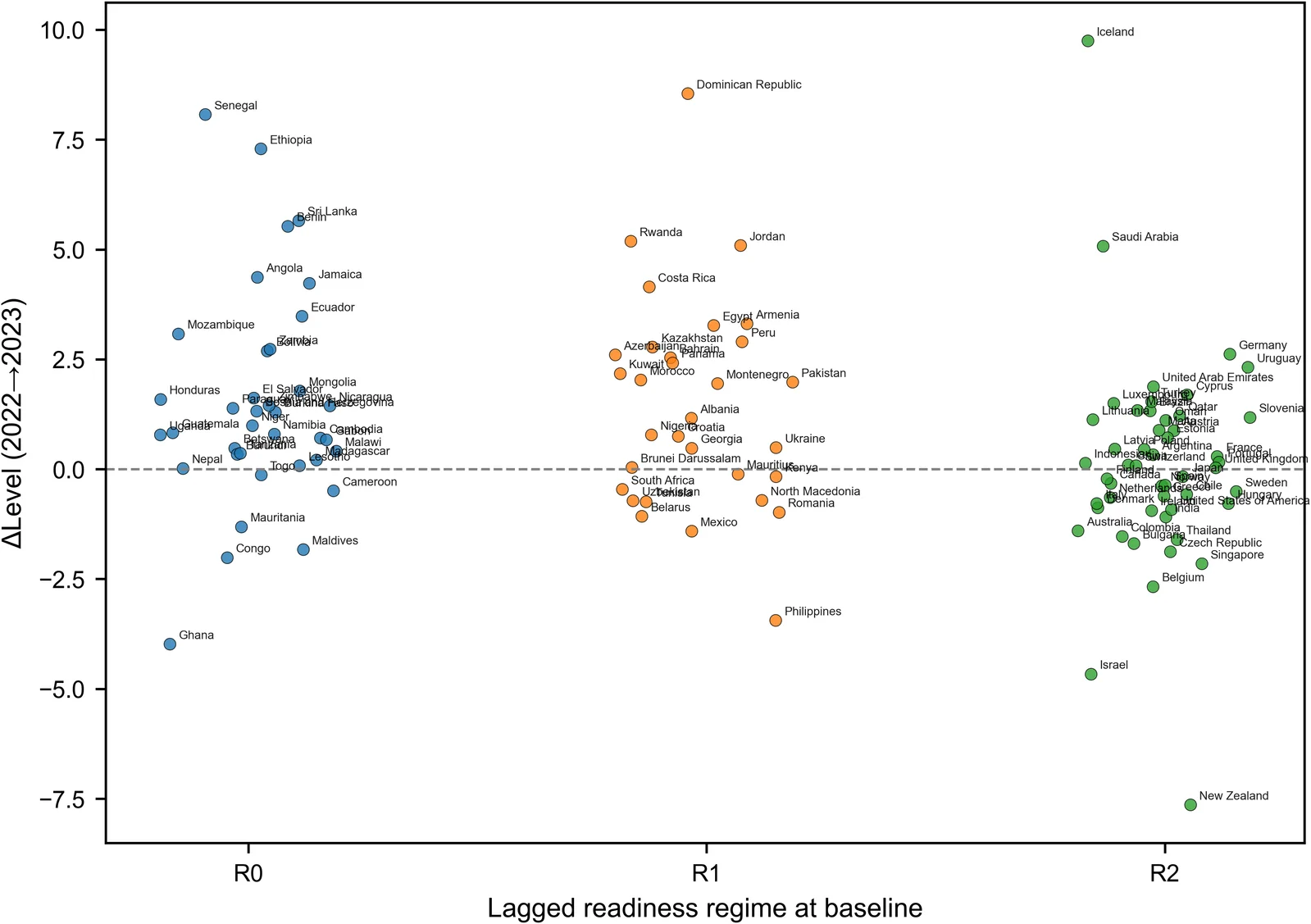
ΔLevel distributions by Regime (W1): Trajectory spread within each stratum.

Three descriptive patterns follow under the lagged specification (Tables 6 and 7). In W1, R2 combines near-flat mean ΔLevel with perfect retention; because the W1 ordering of regime means is mechanical (above), this is not read as a plateau effect. R1 retains every member in W1 while improving, but is the least retained stratum in W2 (Table 6). R0 improves yet is the only mobile stratum in W1 (Table 6), and country-level changes within R0 remain heterogeneous despite its positive mean change. Country-level extremes per regime (Senegal and Ghana in R0; Dominican Republic and Philippines in R1; Iceland and New Zealand in R2) reinforce these patterns; distributions are shown in Fig 8.

#### 5.3.2. Dispersion patterns.

Within-stratum variance changes are treated as exploratory patterns rather than confirmed findings. In W1, R0 variance rises and R2 variance falls; in W2, R0 and R1 rise and R2 reverses (Table 7). No W1 paired-bootstrap interval excludes zero; in W2 the R0 and R1 intervals do (Table 7), although they are not corrected for multiplicity and the six regime-by-window comparisons were not prespecified as a family. Because the seven W1 movers changed W2 membership, the comparison was repeated with 2022 membership fixed: the R1 increase holds, while part of the R0 increase reflects re-composition (Table 7 note). The direction at low readiness is consistent across windows; at high readiness it is not.

### 5.4. Regime transition structure

To address RQ3, this section describes inter-regime transition patterns across the two windows. Zero skip-a-level transitions occur in both windows: all changes are between adjacent strata. Because regimes are contiguous level bands, a skip would require a one-year change crossing the entire middle band: the gap between the highest R0 and the lowest R2 score at the start of each window is 19.03 points (W1) and 20.87 points (W2), larger than any observed one-year change (14.31 points). The zero-skip result is therefore consistent with the documented persistence rather than independent evidence of ordered mobility; a marginal-preserving permutation expects 28.36 skips in W1 and 25.60 in W2 (p = 0.0001), but this null is not proximity-constrained and overstates expected skips.

A common objection is that adjacent-only movement reflects regime construction under limited score variance. The permutation design speaks only to part of this: the null preserves regime sizes but not score proximity, allowing unconstrained reassignment (e.g., R0 → R2) and yielding many expected skips, so the observed zero departs from label-independence. Because the null ignores score proximity, however, it cannot separate ordered mobility from persistence itself: under strong persistence, adjacent-only movement is expected by construction, while a proximity-constrained null would build that persistence into the benchmark and be uninformative. Neither null yields a clean test, which is why the zero-skip regularity is reported descriptively and classified as consistent with persistence rather than independent evidence of ordered mobility (Table 5).

Movement is asymmetric and upward-dominated. In W1, all 7 movers rise from R0 to R1 (Benin, Botswana, Ecuador, Jamaica, Mongolia, Senegal, Sri Lanka), with R1 and R2 fully retained. In W2, 9 countries rise (four R0 → R1: Ghana, Mauritania, Paraguay, Zambia; five R1 → R2: Jordan, Peru, Philippines, Romania, Ukraine) and 3 fall: Botswana and Jamaica (R1 → R0) and Argentina (R2 → R1). No skip transitions occur in either window (Fig 9). Per-regime retention with Wilson 95% CIs for both windows is given in Table 6.

**Fig 9:**
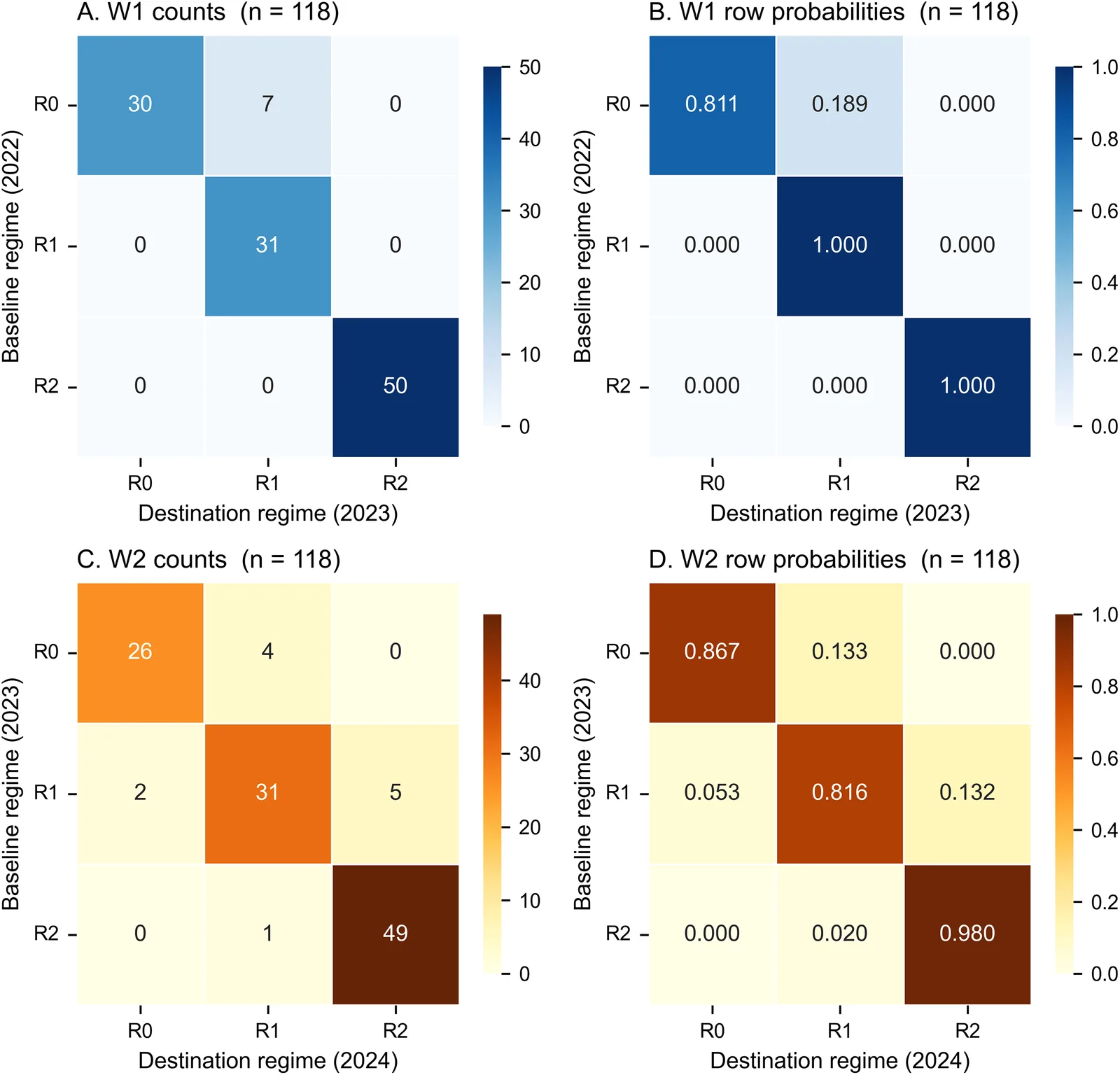
Regime transition matrices: Counts and probabilities for W1 and W2.

Chi-square tests confirm non-random transitions in both windows (Table 5), reflecting persistence rather than a substantive finding. The transition model (fitted on the contemporaneous comparison-variant transitions) assigns most importance to Level (0.832), consistent with regime membership being largely Level-defined; its in-sample accuracy (1.000) reflects the forest fitting its training data and is not informative.

### 5.5. Robustness checks

Under the common held-out protocol the linear specifications track the AR(1) baseline while the flexible ones do not (Table 2). Regime stability is high but not invariant: at k = 3 the primary lagged specification is the more stable in both windows (TSR 0.9407 in W1 and 0.8983 in W2, against 0.9068 and 0.8729 for the contemporaneous variant, whose 2024 assignment reuses the last available change). TSR rises as k falls (Fig 10), and internal validity indices for the comparison variant do not single out k = 3: silhouette and Davies–Bouldin favour k = 2, Calinski–Harabasz favours k = 4. The lagged and contemporaneous assignments differ materially (ARI = 0.4751). Clustering on Level alone reproduces the primary partition exactly (ARI = 1.0000; 0 of 118 countries reassigned), so the primary partition is equivalent to a three-band split on Level and TSR measures retention within these bands; k-means is retained so that the band cut-points are set by the data rather than by arbitrary tertiles. Clustering on standardised features changes 30 of 118 assignments (ARI = 0.5120), so the strata are Level-driven under the chosen metric, not scale-invariant. Together, deviations (Table 3), pillar attribution (Table 4), and regime trajectories (Tables 6–7) provide the empirical basis for the stratum-conditioned follow-up mapping developed in Section 6.

**Fig 10:**
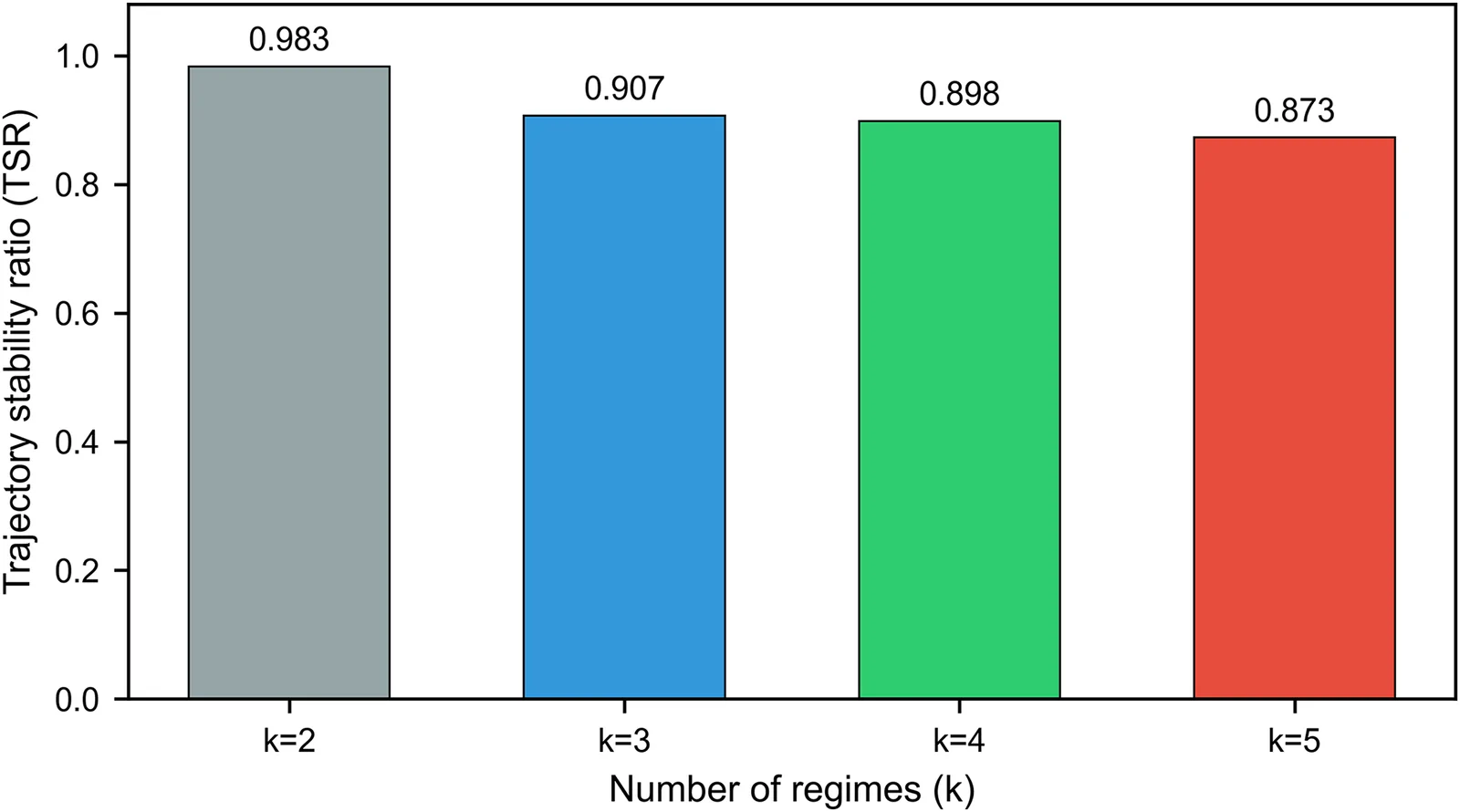
Regime granularity sensitivity: W1 trajectory stability ratio across k (Comparison Variant).

## 6. Discussion and implications

Returning to the organising question (when does a year-to-year change warrant additional analytical attention?), the central finding is that most of the observed variation in published national AI readiness scores is consistent with short-horizon persistence, yet ranking-based interpretation can treat year-to-year changes as performance signals. The framework replaces the implicit rule of “score changed → act” with a structured filter: is this change unexpected given the prior score, and which pillar changed most? The readiness stratum then conditions how a flagged case is read. By construction the rule flags about one in five country-year observations; where it does, the framework identifies the largest normalised pillar change and the stratum-conditioned monitoring focus. This section develops Step 5: translating the results into theoretical interpretation, operational guidance, and research implications.

Existing approaches fall into predictive models optimising forecast accuracy and causal analyses identifying structural determinants [23]; longitudinal change models [31,32] provide established methods for modelling change conditional on prior state. The framework adapts that logic to composite-index constraints (collinearity, short panels, heterogeneity) with a diagnostic, non-causal purpose: screening departures, not forecasting or mechanism estimation.

The study also contributes to development-measurement theory by formalising composite indices as short-horizon persistence systems: state estimates rather than performance measures, whose analytically relevant information resides largely in deviations from expected trajectories. The logic (estimate persistence, analyse residuals, attribute deviations, contextualise by regime) may generalise to other composites built on slow-moving structural variables, from governance to innovation capacity: the question shifts from how ready a country is to whether it is moving as expected given its starting point.

### 6.1. Why High R² is a diagnostic asset: Residuals as the diagnostic unit

Level dominates prediction, regimes are largely Level-defined, and transitions are Level-driven. This apparent tension resolves because the framework’s diagnostic value depends on strong persistence. When nearly all variation in scores is explained by the prior score, the residual becomes the quantity screened for departures from expected persistence. The framework therefore functions as a screening tool for deviations already observed in published annual scores, rather than as a forecasting system. The residual is the diagnostic unit because it is what the persistence baseline leaves unexplained, not because it is itself predictable.

The results are consistent with, but do not test, path-dependence theory: zero skip-a-level transitions are consistent with strong persistence rather than independent evidence of a path-dependent constraint (Section 5.4), and all W1 mobility originates in R0. The framework provides the observational pattern against which path-dependence predictions can be assessed, not causal mechanisms.

### 6.2. Rethinking attribution in AI readiness: Governance as a candidate enabling condition

Government’s position as the pillar most frequently selected by the attribution rule invites a distinction we propose between enabling conditions and scaling dynamics: in the GARI structure as documented by the index [1–4], Government covers regulation, institutional capacity and administrative reform while Technology Sector covers ecosystem depth, R&D and adoption.

Governance investments (AI strategy, data governance, digital government reform, administrative capacity) may be reflected more rapidly in the Government pillar, whereas Technology Sector scores reflect private ecosystem depth that accumulates gradually; the variance gap between the two pillars (Table 4 note) is consistent with this, although it may also reflect how each pillar is constructed. Government’s more frequent selection among short-horizon deviations may therefore reflect responsiveness within the measurement interval rather than ultimate importance; this reading is not tested here. The W2 Technology cases, concentrated in R0 rather than R2, are consistent with larger Technology-pillar movements among lower-readiness cases in that window, but further windows are required to determine whether the pattern is persistent or period-specific.

Whether governance monitoring functions as an earlier warning signal than technology monitoring is a hypothesis these data motivate but cannot establish; it requires external validation against outcomes observed independently of the index. A caveat applies: positive flagged composite residuals for which Government is the leading normalised pillar change may reflect genuine reform or strategic indicator optimisation (e.g., announcing policies without implementation). The framework identifies deviations; qualitative assessment is required to determine whether a deviation reflects substantive change, strategic indicator behaviour or index revision.

### 6.3. Regime-conditioned development: Why uniform interpretation misleads

Although cross-regime differences in mean trajectory change are mechanical (Section 5.3.1), identical score changes do not imply comparable structural conditions: the case for regime-conditioned interpretation rests on the structural heterogeneity documented in the literature, not on the trajectory tests [21,24]. R0 and R2 countries improving by the same amount face different constraints and volatility; treating them as comparable can misdirect diagnostic follow-up. R0 improves on average and is the only mobile W1 stratum (Table 6), yet most of its countries gain without advancing. R0 dispersion rises in both windows, with a paired interval excluding zero only in W2 (Section 5.3.2); longer panels could test whether this reflects bifurcation.

R1 retains every member in W1 but is the least retained stratum in W2 (five rises to R2, two falls to R0; Table 6 and Fig 9), so its stability is window-specific. Dispersion differs by measure and the two should not be conflated: R1 carries the highest baseline score volatility (Table 6), while R0 shows the widest spread of trajectory change (Table 7).

R2 shows near-flat W1 mean ΔLevel with perfect retention, but the W1 ordering of regime means is mechanical (Section 5.3.1) and W2 shows near-complete retention with positive mean ΔLevel, so no plateau or saturation interpretation is supported. Variance patterns are mixed (falling in W1, consistent with convergence, but rising in W2; Table 7), so the convergence pattern is not yet stable. With two windows it is not possible to distinguish structural plateau from transient adjustment, and because the index is re-methodologised across editions (Section 4.1), the W2 non-recurrence of regime differentiation may partly reflect measurement non-invariance.

### 6.4. Adjacent-only mobility consistent with persistence

Zero skip-a-level transitions are observed in both windows: all regime changes occur between adjacent strata. As Section 5.4 shows, this is readily explained by strong persistence and contiguous Level-defined strata [19,21], so it is reported descriptively. Large gains occur both within regimes (Dominican Republic in R1, Iceland in R2) and across them (Senegal and Sri Lanka, R0 → R1 in W1; Mauritania and Ghana, R0 → R1 in W2), so score gain and stratum change are distinct quantities and are not interchangeable as monitoring targets.

### 6.5. Diagnostic follow-up: The Regime-Pillar Matrix

Table 8 maps the (pillar, stratum) pair to a monitoring focus, completing Step 5; Algorithm 1 operationalises the diagnostic sequence. The matrix is a follow-up aid, not prescription: it identifies which readiness dimension to examine given regime and deviation source, and contextual evidence is required before any cell is read as a policy conclusion.

**Table 8:** Regime-pillar diagnostic follow-up matrix.

Regime	Leading Pillar	Monitoring Focus	Cases led (W1; W2)
R0	Government	Governance change at low readiness	6 of 10; 4 of 9 (mostly accelerations)
R0	Data & Infra.	Data and infrastructure movement at low readiness	2 of 10; 1 of 9 (all decelerations)
R0	Tech. Sector	Technology-led movement at low readiness	2 of 10; 4 of 9 (illustrative)
R1	Government	Governance change in the middle stratum, in either direction	3 of 7; 5 of 13
R1	Data & Infra.	Data ecosystem and institutional capacity	4 of 7; 7 of 13
R1	Tech. Sector	Technology-led movement in the middle stratum	0 of 7; 1 of 13 (insufficient)
R2	Government	Governance change at high readiness, in either direction	3 of 7; 2 of 2
R2	Data & Infra.	Data and infrastructure change at high readiness	2 of 7; 0 of 2 (illustrative)
R2	Tech. Sector	R&D, sectoral adoption and innovation ecosystem	2 of 7; 0 of 2 (both accelerations)

*Note.* A diagnostic follow-up aid, not a decision rule: each cell identifies what to examine next, and no cell is causally validated. Each row applies to a flagged observation (residual beyond the flagging band) whose leading normalised pillar change is the listed pillar; the last column gives the flagged cases it leads in that stratum (Table 4). R1 and R2 entries rest on small counts and are provisional pending replication; R0 attribution also rests on modest counts and is indicative. W2 counts by stratum are not variance-adjusted (Section 5.1.2).

### 6.6. Algorithm 1: Diagnostic procedure

The procedure formalises the diagnostic logic for operational use. Algorithm 1 gives the full listing with calibration requirements: its Step 1 covers framework Steps 1–2 (Expect, Detect), Step 2 corresponds to framework Step 3 (Explain), Step 3 to Step 4 (Contextualise) and Step 4 to Step 5 (Interpret & Investigate). Residual thresholds and pillar standard deviations are window-specific, and regime centroids baseline-specific; the W1–W2 shifts in residual thresholds (Section 5.1.2) and pillar standard deviations (Table 4 note) can change which cases are flagged and which pillar leads. In its present form the procedure is a post-release screen: each window’s thresholds are computed from that window’s residual distribution, so prospective use would require thresholds calibrated on earlier windows.

**Algorithm 1: Diagnostic Procedure for Country-Year AI Readiness**

***Input:***

$S_{t-\text{2}},\;S_{t-\text{1}},\;S_{t}$ *pillar scores (Gov, Tech, Data) at t − 1, t; baseline-year scores for the regime centroids (Step 3)*

***Step 1: Persistence (detect deviation)***

• *Estimate AR(1) on your panel: ${\hat{S}}_{t}=\hat{\alpha }+\hat{\beta }\;S_{t-\text{1}}$*

• *Residual:* $\varepsilon_{t}=S_{t}-{\hat{S}}_{t}$

*• If* $P_{\text{1}\text{0}}\leq \varepsilon_{t}\leq P_{\text{9}\text{0}}$ *→ unflagged (no deviation-triggered follow-up)*

• *If* $\varepsilon_{t}>P_{\text{9}\text{0}}$ *→ unexpected acceleration → Step 2*

• *If* $\varepsilon_{t}<P_{\text{1}\text{0}}$ *→ unexpected deceleration → Step 2*

*• (Thresholds* $P_{\text{1}\text{0}}$ *and* $P_{\text{9}\text{0}}$ *are the 10th and 90th percentiles of your residual distribution;*

*recompute for each dataset/window)*

***Step 2: Attribution (identify leading pillar change)***

• $z_{{\;}_{\text{GOV}}}=\Delta {Gov/\sigma }_{GOV}$

• $z_{\text{Tech}}=\Delta {Tech/\sigma }_{Te\text{ch}}$

• $z_{{\;}_{\text{Data}}}=\Delta {Data/\sigma }_{\text{Data}}$ *(σ = cross-country SD of that pillar’s annual change within the window)*

• *Leading pillar =* $\text{arg}\;\text{max}\left(\left|z_{\text{Gov}}\right|,\left|z_{\text{Tech}}\right|,\left|z_{\text{Data}}\right|\right)$

• *Ties (rare with continuous σ-normalised scores) resolve by fixed order: Government, Technology, Data*

***Step 3: Regime (contextualise)***

*• Assign regime using k-means (k by interpretability; TSR descriptive) on {Level, Volatility} (*$\text{Level}=S_{t-\text{1}}$; $\text{Volatility}=\text{SD}\left(S_{t-\text{2}},S_{t-\text{1}}\right)$*), with centroids fitted on the baseline-year cross-section only and applied to subsequent years without re-estimation (Section 4.3)*

***Step 4: Follow-up (investigate)***

• *Map (Regime, Leading pillar) → monitoring focus* (Table 8)

***Output:***

($\varepsilon_{t}$*, Leading pillar, Regime, Monitoring focus)*

*Calibration (must recompute for each dataset/window)*

*• Estimate* $\hat{\alpha }$ *and* $\hat{\beta }$ *from your panel*

*• Recompute residual thresholds (90th, 10th percentiles)*

*• Compute pillar standard deviations* $\sigma_{\text{Gov}}$*,* $\sigma_{\text{Tech}}$*,* $\sigma_{\text{Data}}$

*• Per dataset only: fit regime centroids once on the baseline year and hold them fixed for later windows*

• *(Parameter shifts across windows can change classification and monitoring focus)*

*Structural Rules*

• *1. Interpret changes using persistence-conditioned residuals rather than raw score changes alone*

• *2. Do not prioritise any pillar a priori; use the σ-normalised arg max from Step 2*

• *3. Compare within regimes (Level-defined strata), not across the full ranking or by income*

• *4. Observed transitions were adjacent in these windows; do not treat this as a structural constraint*

### 6.7. Addressing the four analytical gaps

Section 3.5 identified four gaps; each is directly addressed.

**Gap 1:** No residual audit for composite persistence failure. The study provides a full AR(1) residual audit for the Oxford GARI (2021–2024), identifying 24 primary-threshold deviations per window and attributing them to leading normalised pillar changes, with Government the pillar most frequently selected by the attribution rule in both windows (Table 4; Section 5.1.3), findings that reweighting studies [15] cannot produce.

**Gap 2:** No held-out developmental alignment check. Five-fold cross-validated alignment with World Bank income groups (Section 5.2.2) provides an external consistency check beyond within-family index comparisons [14].

**Gap 3:** No temporal within-stratum variance analysis. Within-stratum variance changes are reported across regimes and windows (Table 7; Section 5.3.2) with paired bootstrap intervals. Under the paired bootstrap the W2 increases for R0 and R1 exclude zero (R1 robustly so when 2022 membership is held fixed); the W1 changes and the R2 changes do not, so the direction at high readiness remains unresolved.

**Gap 4:** No regime transition description with inferential transparency. Full 3 × 3 transition matrices, Wilson confidence intervals, and permutation tests are reported (Section 5.4; Table 6; Fig 9). Zero skip-a-level transitions recur in both windows (Section 5.4), a pattern consistent with persistence rather than a structural impossibility [19,21].

### 6.8. Limitations

Seven limitations bound inference; Table 9 states each with its evidential consequence.

**Table 9:** Limitations and inferential boundaries.

Limitation	Description	How it bounds inference
Two transition windows (partial resolution)	2022 → 2023 (W1) and 2023 → 2024 (W2) provide two consecutive transition windows	Among transition patterns, only the zero-skip regularity recurs in W2; mobility differs between windows (7 upward moves in W1; 9 upward and 3 downward in W2); further annual windows needed for long-run inference
Index architecture conditionality	Results reflect Oxford GARI pillar structure, weights, and normalisation	May not replicate on Stanford AI Index or NRI; cross-index replication essential before generalising
Short panel	Four years insufficient for causal inference or long-run trend analysis	Framework designed for short panels; five or more years would enable stronger variance tests
Within-stratum variance dynamics	Longer panels could test whether dispersion systematically increases at lower readiness and contracts at higher readiness	The present two-window panel is insufficient to establish such dynamics; W2 strata also change membership after the W1 moves (Section 5.3.2)
No causal identification	Framework diagnoses where and how deviations occur, not why	Government is the pillar most frequently selected by the attribution rule; no causal claim is made or implied
Index methodology changes across editions	Oxford GARI was revised across editions (Section 4.1), 2021–2024	Cannot distinguish genuine capability change from measurement change
Heteroskedastic residuals (W2)	Residual dispersion falls with readiness in W2 (Breusch–Pagan p = 0.017; Section 5.1.2)	A single global band flags fewer high-readiness cases; the stratum distribution of flags is not variance-adjusted

### 6.9. Future research

**Longitudinal validation.** The primary test is continued replication as new Oxford GARI editions become available. The current four-year panel (two transition windows) partially addresses single-window limits: the adjacent-only mobility pattern recurs, consistent with persistence, and Government remains the pillar most frequently selected by the attribution rule in both windows, with wide uncertainty. Extending to five or more years would improve power for the within-stratum dispersion question, establish skip-transition base rates, and test whether Government’s selection frequency and the W2 Technology concentration in R0 are stable or period-specific. A key extension is external threshold validation: applying W1-calibrated parameters to W2 and comparing classifications against documented reforms, investments, or shocks. This would move from percentile thresholds to externally validated diagnostic cut-offs.

**Cross-index replication.** Findings are currently conditioned on Oxford GARI. Replication on longitudinal composite indices with comparable country-level scores and pillar structure, such as the Network Readiness Index, would test generality; the Stanford AI Index and OECD AI Observatory indicators can provide external context. Cross-index replication would help distinguish patterns that generalise across AI-readiness measures (the persistence structure, adjacent-only mobility and, where a comparable governance pillar exists, Government’s selection frequency) from those specific to the Oxford GARI architecture or the present observation period. The protocol is fixed: estimate AR(1), derive residual thresholds, normalise pillar changes, define regimes via k-means on the strictly lagged {Level, Volatility} features, and test the same patterns. Differences should be traced to pillar architecture, positioning the framework as a transferable methodological template.

Generalisation beyond AI readiness. The logic may extend to other composite indices of slow-moving structural variables: the Worldwide Governance Indicators, OECD Digital Economy Outlook, or Global Innovation Index may exhibit similar persistence–residual structure, and application there would test whether governance-type enabling conditions are associated with larger normalised deviations across domains.

Causal identification and qualitative validation. The framework detects where the published score departs from the fitted persistence expectation and which normalised pillar change is largest, not why the departure occurred. Event studies of governance reforms could test whether particular policies generate observed pillar movements, and qualitative case studies of flagged countries would provide institutional context beyond the algorithm’s scope.

## 7. Conclusion

This paper addresses a central limitation of AI readiness measurement: raw composite-score changes do not distinguish routine persistence from departures that warrant additional diagnostic attention. Treating composite scores as short-horizon persistence systems, the five-step framework (Expect, Detect, Explain, Contextualise, Interpret & Investigate) shifts the analytical focus from levels to deviations; it is applied here to Oxford GARI data across 118 countries and two consecutive transition windows.

Three findings anchor the contribution. First, persistence is a diagnostic asset: with 97.9% of W1 score variation explained by prior scores, residuals become the screening unit, while held-out validation shows that no flexible specification improves on that baseline. Second, Government is the pillar most frequently selected by the attribution rule among flagged deviations, with wide uncertainty (50.0% in W1, 45.8% in W2), with the ranking threshold-dependent (Data & Infrastructure leads under a 20th/80th rule). Third, all observed regime moves were adjacent: zero skip-a-level transitions in both windows, interpreted as consistent with persistence rather than independent evidence of constrained movement.

Methodologically, the two-window design distinguishes recurring from period-specific patterns: persistence structure, adjacent-only mobility, and the leading-pillar pattern recur, while attribution shares vary. The W1 regime differentiation is consistent with a mean-reversion artefact under the below-unity AR(1) slope, a pattern that itself illustrates the framework’s central claim: in persistence-dominated systems, apparent findings can be persistence in disguise, so explicit trajectory conditioning is required even of the analyst.

The framework is conditioned on the Oxford GARI structure, relies on a short panel insufficient for causal inference, does not identify mechanisms, treats residuals as departures in published GARI scores whose editions are not measurement-invariant, leaves some regime-level patterns resting on small counts, and derives thresholds internally rather than by external validation; replication across indices and longer horizons is therefore necessary for generalisability.

The operational implication is direct: analysts supporting ministries and development organisations could apply the framework (Algorithm 1) annually to each new GARI release, separating the roughly four-in-five observations that the signed-decile rule leaves unflagged by construction from the one-in-five it flags, and mapping the latter to pillar- and stratum-conditioned follow-up. The contribution is not improved prediction of where countries will be, but disciplined identification of when a change warrants attention, which pillar changed most, and what to examine next given the observation’s readiness stratum. Composite indices can be more than benchmarking tools; this framework shows how.
